# Supplementary material for: Automatic Posture and Movement Tracking of Infants with Wearable Movement Sensors
Source: Sci Rep. 2020 Jan 13;10:169. doi: 10.1038/s41598-019-56862-5 (PMC6957504; doi:10.1038/s41598-019-56862-5)
Supplement: Supplementary file 1 — supplementary informations. [file 41598_2019_56862_MOESM1_ESM.pdf]

SmartJumpsuit video annotation guidelines, v2.2

Manu Airaksinen

1 Track A: Posture

Definition: **The overall posture of the baby**

1. Look for change of posture events from video/signals.
2. Roughly approximate label boundary from video.
3. Look for accurate boundary cues from the signals of the prime mover limb(s)
4. All time instants (except for out of screen/missing sensor data) should have exactly one active category

Tip: boundary should be at half-way point of the transition movement

**prone:**	- When baby is, in general, on their belly (navel on the ground, includes push-up position)<br>- Gravity vectors in accelerometer signals should be roughly symmetrically aligned between left-right axis
**supine:**	- When baby is, in general, on their back (lower back on the ground)<br>- Gravity vectors in accelerometer signals should be roughly symmetrically aligned between left-right axis
**side L/R:**	– When baby is primarily on Left/Right side<br>- Clear case: If one side's hand and knee is off the ground<br>- Ambiguous case: asymmetrical weight distribution in L/R axis AND/OR clearly asymmetrical hip placement<br>- Look for asymmetrical gravity vectors in L/R axis if unsure based on video (i.e., "hand and knee off the ground" is not precisely met)
**crawl_posture:**	- When baby is supported by their hands AND (knees OR feet)<br>- Includes "plank" position, but not arm-supported crawl position ("cobra pose")<br>- Precise boundary cues from accelerometer gravity vectors
**on_lap:**	- Outlier category for when baby is on lap/being handled by a caregiver

2 Track B: Movement

Definition: **Movement modes or patterns that commonly result in the change of the baby's position and/or posture in space.**

1. Look for movements in the video/signals
2. Identify the beginning and end of the holistic movement pattern from the waveforms.
3. Each change of posture in track A should be accompanied by a segment in track B encapsulating the posture boundary

Tip: Macro movement typically starts with the "winding" of a prime mover limb that is visible in the waveforms.
- Include this in the movement categories, even though the baby is at this time still "macro stationary"

Tip2: Visible periodic movement patterns containing multiple limbs, but not resulting in macro movement, should be classified as "proto_crawling/jiggling"

**macro_still:**	- (No movement present that results in a change of the baby's position or posture) AND (baby is not in a "crawl_proto/jiggling" mode, e.g. kicking)
**crawl_commando:**	- Crawling that results in forward movement in prone/side/4limbs position that is primarily done by dragging oneself forward<br>- Includes dragging style crawling also within the 4 limbs position
**crawl_4limbs:**	- Crawling on 4 limbs that exhibits clear periodic movement pattern peaks in the waveforms (i.e., movement by correctly "walking" the limbs, not dragging them)
**crawl_proto/jiggling:**	- Category for movement "modes" during all postures that have clear periodic structure and do not result in macro movement<br>- Includes / examples of this category:<br>- During prone/side postures: "proto_crawling", where baby is practicing crawling patterns without actually moving<br>- During supine posture: "kicking", "clapping", "jiggling" (baby moves/flexes multiple limbs periodically similarly to proto crawling)<br>- DOES NOT include:<br>- Purely distal limb movements (e.g., moving of only hands/wrists/feet).<br>- Random/one-off movements of limbs. The baby should be in a "movement mode" that contains at least 2-3 iterations of the pattern within a time frame of at least 2-3 sec.
**pivot L/R:**	– Pivoting results in the change of facing direction of the whole body, without simultaneous forward movement<br>- If forward movement is present alongside pivoting, consider classifying as crawl_commando (depending which you deem as the dominant category)<br>- Pivoting can also result in the change of posture (Track A)
**turn L/R:**	- Turning always results in change of posture along the 'prone' – 'side' – 'supine' axis<br>- L/R defined by looking from baby's view and determining if the direction of rotation is achieved by turning the baby's head Left or Right.

3 Track D: Other

Definition: **Metadata track for marking 'out of screen' and 'handling' events that are not used in the training of the classifier.**

**out_of_screen:**	- If baby is out of screen in the video, and annotation cannot be made.
**handling:**	– If baby is being handled in a way by a caregiver/physiotherapist that is visible in the measurement signals:<br>- Includes / examples of this category:<br>- Lifting, tapping, carrying, lifting of hands/legs, . . .<br>- DOES NOT include (if not visible in the sensors):<br>- Soft touch (soothing, stroking, gently holding hands)
