# Supplementary material for: Automatic Posture and Movement Tracking of Infants with Wearable Movement Sensors
Source: Sci Rep. 2020 Jan 13;10:169. doi: 10.1038/s41598-019-56862-5 (PMC6957504; doi:10.1038/s41598-019-56862-5)
Supplement: Supplementary file 2 — supplementary informations 2. [file 41598_2019_56862_MOESM2_ESM.pdf]

Supplementary Material

Automatic Posture and Movement Tracking of Infants with Wearable Movement Sensors

Manu Airaksinen, Okko Räsänen

Contents

1 Data pre-processing 1

2 Overview of soft labels 1

3 Example of iterative annotation refinement (IAR) 2

4 CNN classifier structure and training 3

5 Recording-level classifier performance with raw labels 4

6 CNN overall performance with raw labels 6

7 SVM overall performance with raw labels 7

8 SVM overall performance with IAR processed labels 8

9 References 8

1 Data pre-processing

The pre-processing of the raw multi-sensor signals was performed with a minimal approach, as in no extra filtering or noise reduction steps were applied to the raw signals. As the most important part of pre-processing, the integrity of the timestamps was ensured by synthesizing an ideal timestamp vector at the target sampling rate (52 Hz), and all raw signals were linearly interpolated to match the synthesized timings at regular intervals. This mitigates the effects of sensor clock jitter and packet loss from further analysis pipeline. With the interpolated signals, the bias in each gyroscope channel was removed by subtracting the mean of the 64-sample frame with the smallest variance within the channel (measured across the entire recording).

2 Overview of soft labels

The annotation given by the human annotators have distinct starting and ending points for each annotated category. The raw annotations for all tracks (posture, movement, metadata) were first converted into a $\hat{\mathbf{v}}_{\mathbf{tr}} = (N, 1)$ sized column vector, where tr is the name of the track and N is the length of the sensor recording in samples. The elements of $\hat{\mathbf{v}}_{\mathbf{a}}$ that were within the boundaries of the starting and ending instants of each annotated category activation were set to a discrete value corresponding to the category. The metadata track (containing categories for carrying and out of screen events) was fused into a binary track (where activation means the activation of either of the annotated classes). The movement and posture tracks were masked with the metadata track by

element-wise multiplication (i.e., $\hat{\mathbf{v}}'_{\mathbf{tr}} = \hat{\mathbf{v}}_{\mathbf{tr}} \odot \hat{\mathbf{v}}_{\mathbf{meta}}$), so that resulting zeros became a new discrete `unknown` category.

Based on the chosen frame length (N_{wl}) and frame skip (N_{hop}), each annotator's signal-length annotation track was windowed into frame-specific sub-vectors $\hat{\mathbf{v}}'_{\mathbf{tr,i}} = \hat{\mathbf{v}}'_{\mathbf{tr}}[(i-1)*N_{hop} : (i-1)*N_{hop} + N_{wl}]$, where i is the frame number of the recording. The discrete annotation c_i was obtained by $c_i = \text{mode}(\hat{\mathbf{v}}'_{\mathbf{tr,i}})$. An exception was if any `unknown` annotations were present at the frame, where the frame was set to `unknown` regardless if it was the mode category or not. The framed labels for each annotator were then gathered together as a column vector $\mathbf{L_a} = [c_1, c_2, \ldots, c_{Nfr}]^T$, where a is the annotator id, and N_{fr} is the number of frames within the recording.

After obtaining the annotator-specific labels for the frames for the three parallel annotators, the labels were converted into a one-hot form. I.e., each annotation was presented by a matrix $L_{oh,a} \in R^{(N_{fr}, N_{cats})}$, where N_{cats} was the number of categories in the track (+1 for the `unknown` category). Each row of $L_{oh,a}$ consists of zeroes except for the index corresponding to the current category, which was set to one. An example of this is presented below:

$$\begin{bmatrix} 1 \\ 1 \\ 2 \\ 5 \\ 3 \end{bmatrix}_{\text{categorical}} = \begin{bmatrix} 1 & 0 & 0 & 0 & 0 & 0 \\ 1 & 0 & 0 & 0 & 0 & 0 \\ 0 & 1 & 0 & 0 & 0 & 0 \\ 0 & 0 & 0 & 0 & 1 & 0 \\ 0 & 0 & 1 & 0 & 0 & 0 \end{bmatrix}_{\text{one-hot}}$$

The final step in obtaining the soft labels was by averaging the one-hot matrices by $L_{oh,tot} = \frac{1}{N_{annot}} \sum_{a=1}^{N_{annot}} L_{oh,a}$, where $N_{annot} = 3$ is the number of parallel annotations. An example of obtained soft labels is presented below, where the three discrete annotation tracks on the left side have been concatenated to form a matrix of dimensions $(N_{fr}, 3)$:

$$\begin{bmatrix} 1 & 1 & 1 \\ 1 & 2 & 1 \\ 2 & 2 & 1 \\ 5 & 3 & 2 \\ 3 & 3 & 3 \end{bmatrix}_{\text{categorical}} = \begin{bmatrix} 1 & 0 & 0 & 0 & 0 & 0 \\ 0.66 & 0.33 & 0 & 0 & 0 & 0 \\ 0.33 & 0.66 & 0 & 0 & 0 & 0 \\ 0 & 0.33 & 0.33 & 0 & 0.33 & 0 \\ 0 & 0 & 1 & 0 & 0 & 0 \end{bmatrix}_{\text{soft}}$$

As with the discrete frames, the presence of the `unknown` category over-rides other annotations, and the column of L_{oh_tot} corresponding to it was removed from the matrix to act as a binary mask for training.

The output layer of the CNN produces output activations for the desired categories that were normalized into a probability distribution with the softmax function, thus making the soft labels directly usable in training.

3 Example of iterative annotation refinement (IAR)

An example of the annotation refinement update for five frames (rows) is given below (hum = human prior; cls = classifier softmax output; iar = IAR result; iar,norm = normalized IAR result):

$$\begin{bmatrix} 1 & 0 & 0 & 0 & 0 & 0 \\ .66 & .33 & 0 & 0 & 0 & 0 \\ .33 & .66 & 0 & 0 & 0 & 0 \\ 0 & .33 & .33 & 0 & .33 & 0 \\ 0 & 0 & 1 & 0 & 0 & 0 \end{bmatrix}_{\text{hum}} \odot \begin{bmatrix} .6 & .2 & .04 & .06 & .09 & .01 \\ .15 & .45 & .2 & .1 & .05 & .05 \\ .3 & .4 & .2 & .1 & .1 & .1 \\ .1 & .5 & .2 & .05 & .1 & .05 \\ .05 & .3 & .4 & .15 & .05 & .1 \end{bmatrix}_{\text{cla}} = \begin{bmatrix} .6 & 0 & 0 & 0 & 0 & 0 \\ .1 & .15 & 0 & 0 & 0 & 0 \\ .1 & .26 & 0 & 0 & 0 & 0 \\ 0 & .17 & .07 & 0 & .03 & 0 \\ 0 & 0 & .4 & 0 & 0 & 0 \end{bmatrix}_{\text{iar}}$$

$$= \begin{bmatrix} 1 & 0 & 0 & 0 & 0 & 0 \\ .4 & .6 & 0 & 0 & 0 & 0 \\ .27 & .73 & 0 & 0 & 0 & 0 \\ 0 & .63 & .25 & 0 & .13 & 0 \\ 0 & 0 & 1 & 0 & 0 & 0 \end{bmatrix}_{\text{iar,norm}}$$

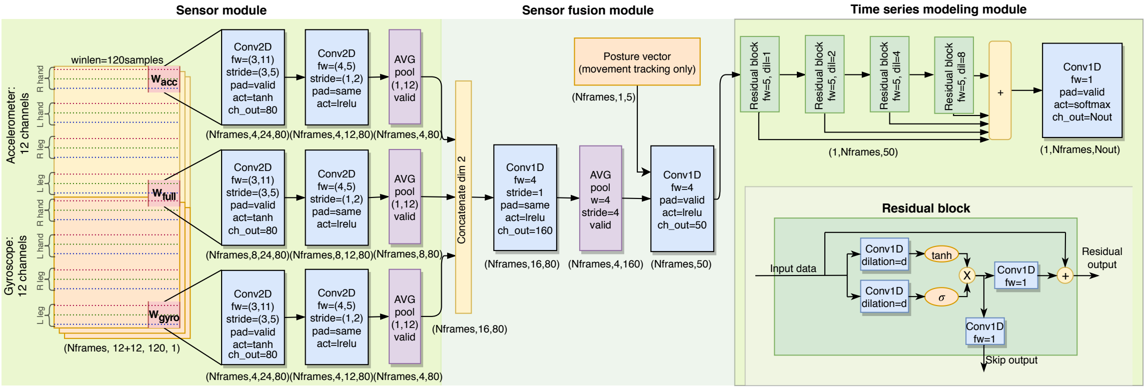

Figure 1: Detailed structure of the utilized CNN model, including filter widths, activation functions, and strides (when differing from 1). Resulting tensor shapes at each parts of the network are also shown.

4 CNN classifier structure and training

The CNN classifier, depicted in Figure 1, takes as an input a framed raw signal tensor of shape (Nframes, Nchans, wlen,1). For our experiments, wlen=120 and Nchans=24 and Nframes is computed by floor((siglen-wlen)/hop)+1, where hop=60. The input signals are scaled by constants (1/100 for gyro, 1/10 for acc) to obtain signal amplitudes close to the range [-1, 1]. The scaling was done to mimic typical min-max normalization so that the relative DC-levels for each channel would stay constant, In addition, dropout with p=0.3 is applied to the input frames during training to mitigate over-fitting, which simultaneously acts as a data augmentation method that boost performance during packet loss. Dropout was also experimented with at the beginning of the time series modeling module, which yielded similar results.

The sensor module components perform dimensionality reduction with 2-dimensional convolutions followed by an average pooling operation, followed by concatenation of their outputs. The sensor fusion module assimilates the learned sensor-level features into latent features representing the entire frame. Finally, the time series modeling module performs gated dilated convolutions over the tensors' frame axis to model the time-characteristics of the frame-level features. The receptive field of the dilated convolution stack is 30 frames (15 future, 15 past, 34.6 seconds) that are used to condition each frame's outputs. The output of the network is a matrix of shape (Nframes,Ncats), which is is normalized at the frame-level by the softmax function.

The training is performed by applying the categorical cross-entropy criterion between the predicted and target labels of the training data. The training mask discussed in Section 2 is used to mask the error for `unknown` -labeled frames. In order to account for the heavily skewed category distributions of the training data (e.g., see Figure 2a of the main article), each frame's error was scaled based on the inverse of the empirical frequency of each class in the training data. The used probabilities for the posture track were: `prone`=0.615, `supine`=0.27,`sideL`=0.035, `sideR`=0.035,`crawl posture`=0.045, and for the movement track: `still`=0.56, `turnL/R`=0.02, `pivotL/R`=0.04, `crawl commando`=0.04, `crawl proto`=0.26.

The training of the CNN was performed with mini-batch stochastic gradient descent with the backpropagation method, using the Adam algorithm [1] with learning rate $= 10^{-4}$, $\beta 1 = 0.9$, and $\beta 2 = 0.999$ to perform the SGD updates. The mini-batch size was 100 consecutive frames, and at the beginning of training, 20% of each recording's batches were randomly selected as the validation set for the early stopping criterion. The training was run for 120 epochs, and the system from the epoch with the highest prediction accuracy on the validation data was selected as the fully trained model.

The code for the CNN classifier was implemented with Tensorflow (v1.8.0), and the LOSO crossvalidation and IAR methods were implemented with Python(v3.6.6)/NumPy(v1.14.5). The code is available at request.

5 Recording-level classifier performance with raw labels

This Section presents results obtained with training the classifiers by the “rar” labels, i.e., the labels obtained from human annotations with majority vote. Figure 2 presents comparison between the CNN (blue) and SVM (red) classifiers trained with raw labels. Figures 3 and 4 present CNN-specific results comparing performance between systems trained with the Raw labels (blue) and IAR-processed labels (green) as measured by the “full human agreement” (3/3) set, and the “all frames” set. Figure 5 otherwise reproduces Figure 8 of the main manuscript, but the CNN classifier has been trained with the raw labels.

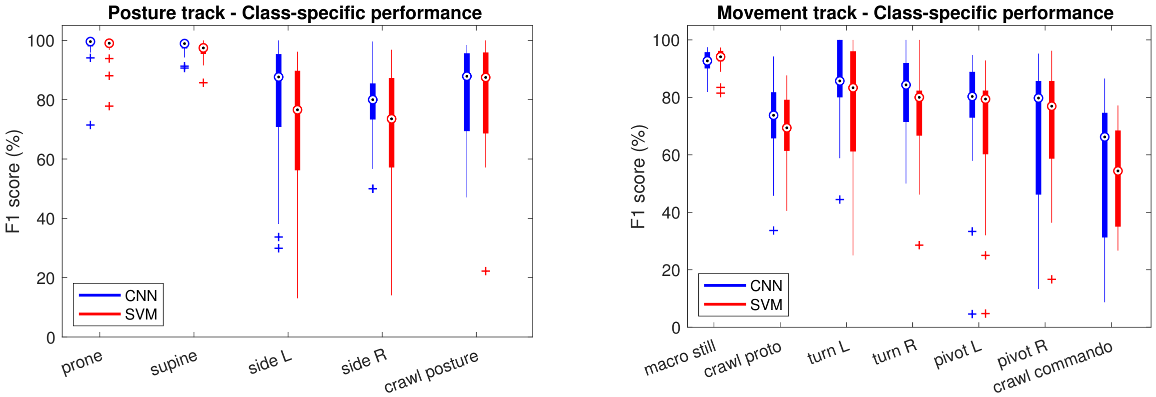

Figure 2: Class-specific F1-score box plots for individual recordings for the Posture and Movement tracks using the CNN and SVM classifiers trained with raw labels.

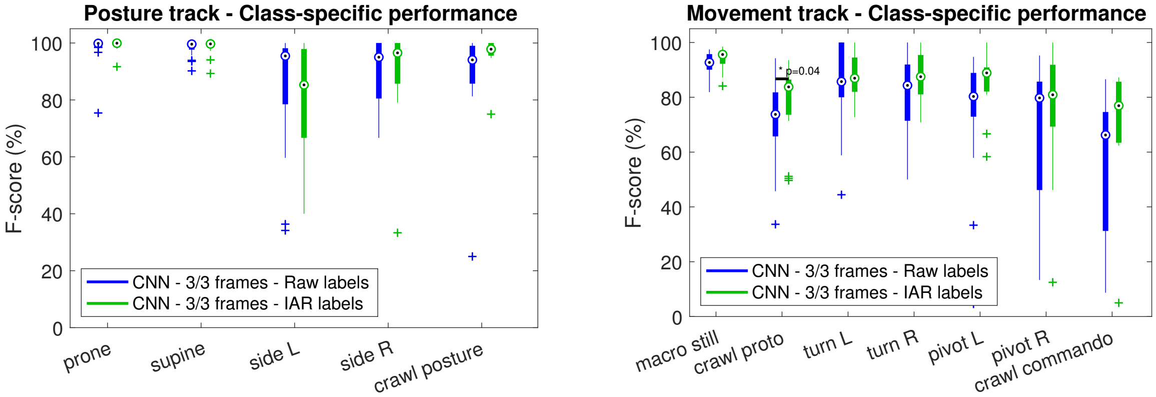

Figure 3: Comparison of CNN performance on “full human agreement (3/3) frames set” when the system is trained with raw (blue) and IAR processed (green) labels.

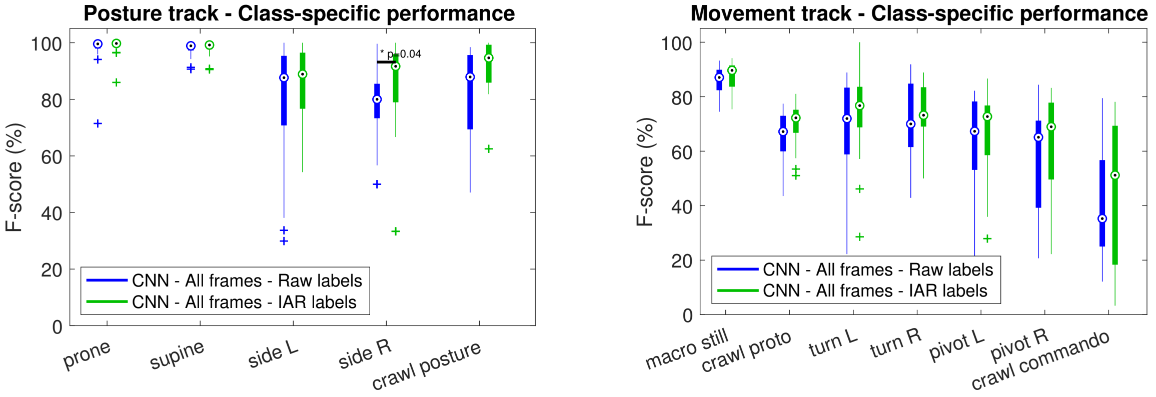

Figure 4: Comparison of CNN performance on "all frames set" when the system is trained with raw (blue) and IAR processed (green) labels.

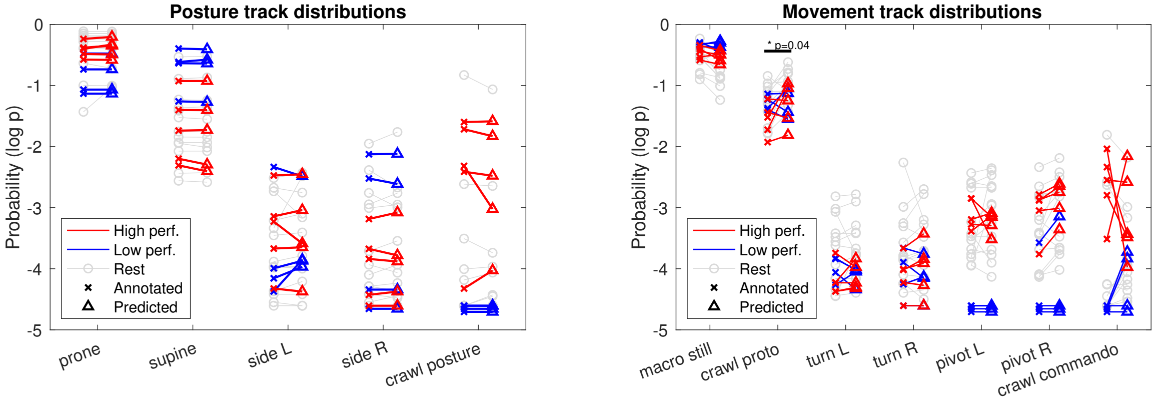

Figure 5: Figure 8 of the manuscript computed with Raw-label trained classifier outputs.

6 CNN overall performance with raw labels

This Section presents the full confusion matrices and their unweighted average metrics for the CNN classifier performance when trained with the raw labels (majority vote from human annotations).

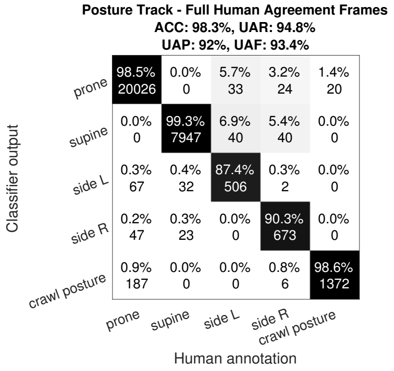

(a) CNN, Raw label training

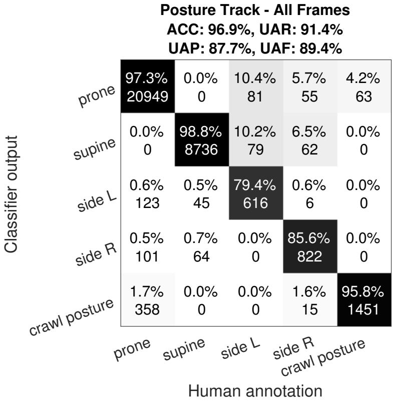

(b) CNN, Raw label training

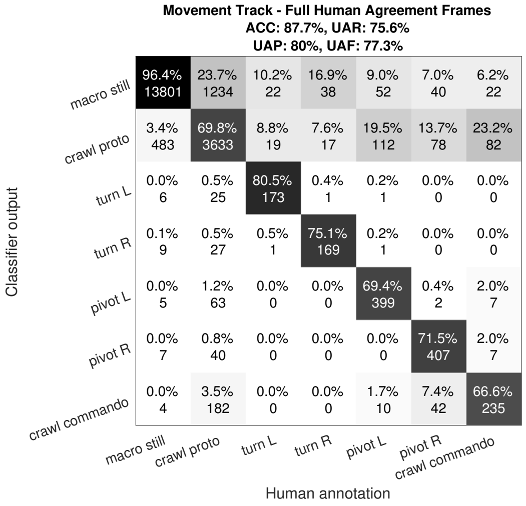

(c) CNN, Raw label training

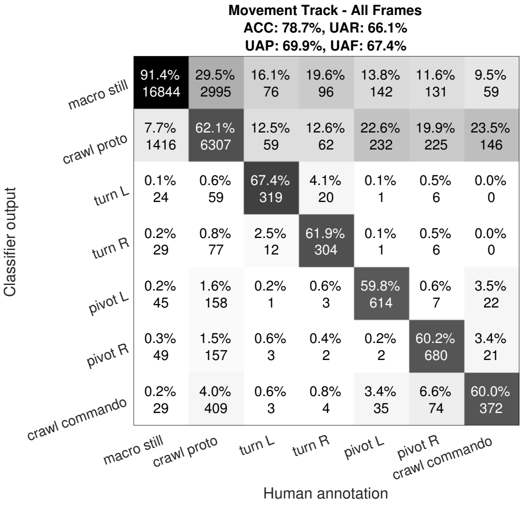

(d) CNN, Raw label training

Figure 6: CNN confusion matrices of the Posture (a and b) and Movement (c and d) tracks obtained from LOSO cross-validation of the (a and c) full annotation agreement subset and (b and d) complete data set. Class-specific precision (row) and recall (column) values are shown, and the average accuracies are reported in the bottom-right corners.

7 SVM overall performance with raw labels

This Section presents the full confusion matrices and their unweighted average metrics for the SVM classifier performance when trained with the raw labels (majority vote from human annotations).

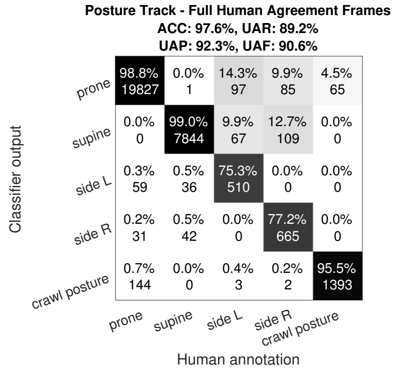

(a) SVM, Raw label training

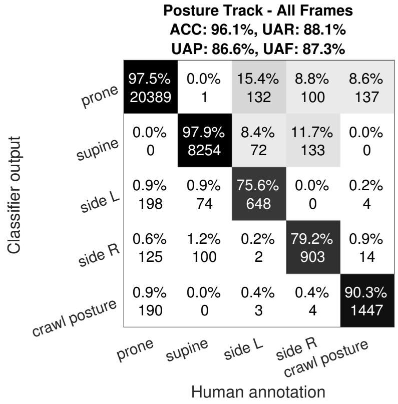

(b) SVM, Raw label training

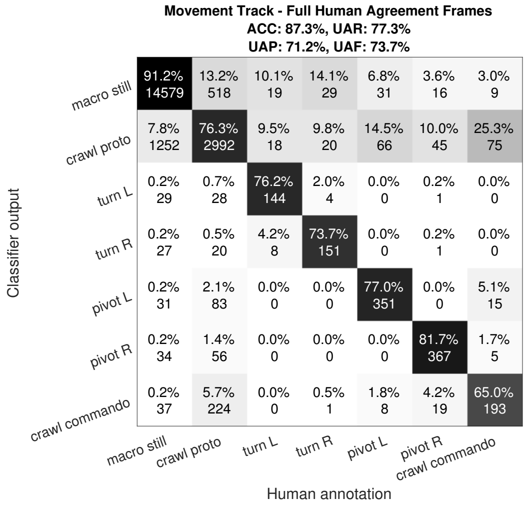

(c) SVM, Raw label training

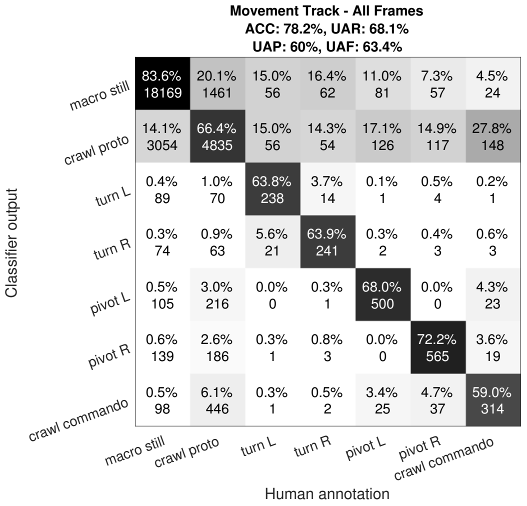

(d) SVM, Raw label training

Figure 7: SVM classifier confusion matrices of the Posture (a and b) and Movement (c and d) tracks obtained from LOSO cross-validation of the (a and c) full annotation agreement subset and (b and d) complete data set. Class-specific precision (row) and recall (column) values are shown, and the average accuracies are reported in the bottom-right corners.

8 SVM overall performance with IAR processed labels

This Section presents the full confusion matrices and their unweighted average metrics for the SVM classifier performance when trained with the IAR-processed labels.

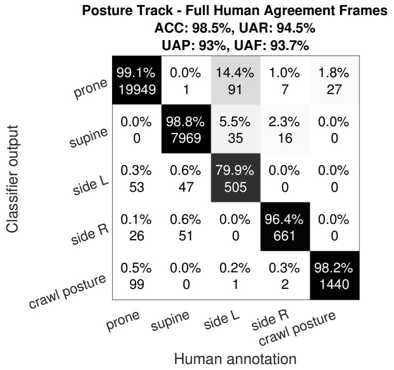

(a) SVM, IAR label training

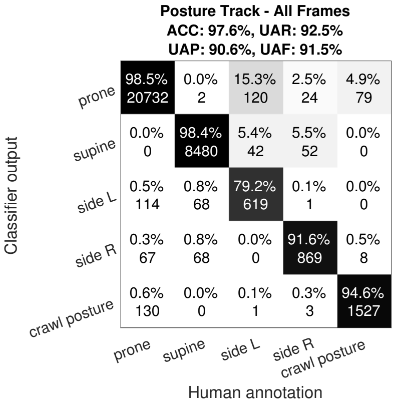

(b) SVM, IAR label training

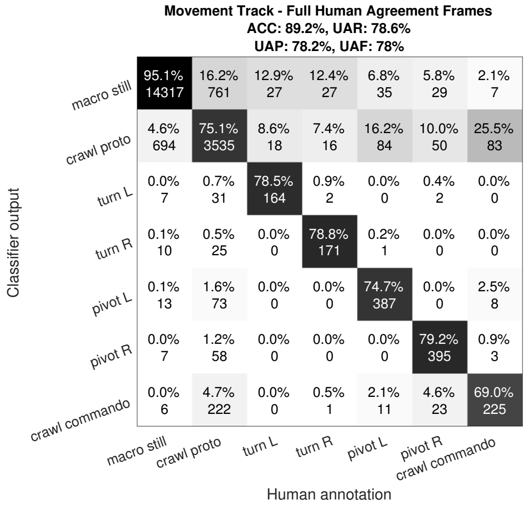

(c) SVM, IAR label training

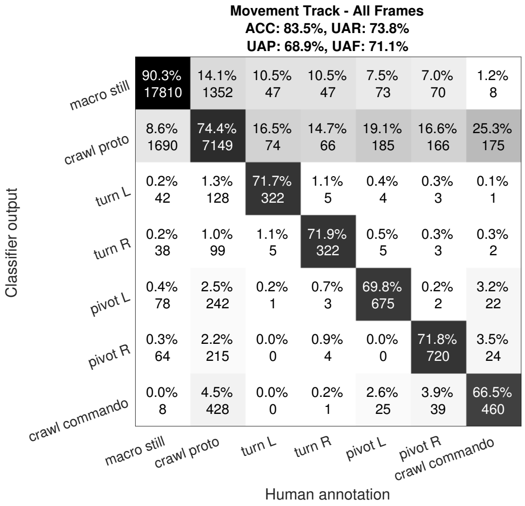

(d) SVM, IAR label training

Figure 8: SVM classifier confusion matrices of the Posture (a and b) and Movement (c and d) tracks obtained from LOSO cross-validation of the (a and c) full annotation agreement subset and (b and d) complete data set. Class-specific precision (row) and recall (column) values are shown, and the average accuracies are reported in the bottom-right corners.

9 References

[1] D. Kingma, J. Ba: *"Adam: A Method for Stochastic Optimization"*, arXiv:1412.6980, 2014
